# Deep learning auto‐contouring of target and organs‐at‐risk on post‐catheter implant CT images for prostate HDR brachytherapy

**DOI:** 10.1002/acm2.70748

**Published:** 2026-08-18

**Authors:** Eric M. Wallat, Joseph B. Schulz, John M. Floberg, Gregory Cooley, Bryan P. Bednarz, Jordan M. Slagowski

**Affiliations:** ^1^ Department of Radiation Medicine University of Wisconsin–Madison Madison Wisconsin USA; ^2^ Department of Medical Physics University of Wisconsin–Madison Madison Wisconsin USA

**Keywords:** brachytherapy, deep learning, prostate cancer, segmentation

## Abstract

**Background:**

Accurate delineation of the prostate and surrounding organs‐at‐risk (OARs) is essential for HDR prostate brachytherapy. Manual contouring on post‐catheter CT images is time‐consuming and prone to variability due to artifacts and anatomical deformation from the implanted brachytherapy catheters.

**Purpose:**

To develop and validate a 3D deep learning autosegmentation model for prostate and OAR contouring on post‐catheter CT images for prostate brachytherapy planning.

**Methods:**

A self‐configuring U‐Net architecture (nnU‐Net) was trained on 206 HDR prostate brachytherapy cases and tested on an additional 36 patients. Structures included prostate PTV, bladder, rectum, and urethra identified from a Foley catheter. Performance was evaluated against a commercially available model using geometric (Dice coefficient (DSC), Hausdorff distance (HD95%), average surface distance (ASD)) and dosimetric metrics (PTV V100%, bladder and rectum D1cc, urethra D0.1cc). Statistical significance was assessed using paired Wilcoxon signed‐rank tests.

**Results:**

NnU‐Net achieved superior geometric accuracy versus the commercial model for all structures within slices containing the prostate. For prostate PTV, nnU‐Net yielded DSC = 0.91 +/− 0.04, HD95% = 3.62 +/− 2.23 mm, and ASD = 1.25 +/− 0.66 mm, compared to DSC = 0.78 +/− 0.07, HD95% = 9.52 +/− 4.73 mm, and ASD = 2.97 +/− 0.90 mm for the commercial model. Urethra segmentation was only provided by nnU‐Net (DSC = 0.84 +/− 0.09). Dosimetric differences for nnU‐Net were clinically negligible: ΔPTV V100% = −0.25 +/−2.30%, bladder ΔD1cc = 0.13 +/− 0.75 Gy, rectum ΔD1cc = 0.34 +/− 0.74 Gy, and urethra ΔD0.1cc = −0.03 +/− 0.16 Gy. Differences in PTV V100% were not significant for nnU‐Net (*p* = 0.91) but significant for the commercial model (*p* < 0.001).

**Conclusions:**

A brachytherapy‐specific nnU‐Net model enables accurate autosegmentation of prostate and OARs on post‐catheter CT images, outperforming an EBRT‐trained commercial solution. Minimal dosimetric differences support clinical feasibility and potential workflow improvements in HDR prostate brachytherapy.

## 1 INTRODUCTION

Prostate cancer is the most common malignancy amongst men in the United States, accounting for 15.4% of all new cancer cases with 5‐ and 15‐year relative survival rates at 98% and 97%, respectively.^1^ As prostate cancer survivors often live many years post‐diagnosis, the management and prevention of treatment‐related side effects becomes increasingly important. Treatment options for localized prostate cancer include active surveillance, radical prostatectomy, external beam radiation therapy (EBRT), and brachytherapy (BT). High‐dose‐rate (HDR) prostate brachytherapy, in particular, is frequently used as a boost in combination with EBRT or as monotherapy due to its ability to deliver precise, escalated doses in a controlled manner.^2^, ^3^, ^4^

Accurate delineation of the prostate and nearby organs‐at‐risk (OARs) is critical for HDR brachytherapy planning. Contouring errors can lead to suboptimal dose coverage or unintended exposure of OARs, directly impacting treatment quality and patient safety. However, manual segmentation is time‐consuming and subject to inter‐ and intra‐observer variability, which can be exacerbated in the brachytherapy setting where patients are under anesthesia and planning is done on‐the‐fly using images that contain artifacts and anatomical deformations due to applicators, fiducial markers, and urinary catheters.^5^

Artificial intelligence (AI)‐based autosegmentation has emerged as a promising solution to these challenges, offering the potential to reduce variability, improve efficiency, and enhance reproducibility in treatment planning. A recent review by Chen et al. emphasized that while AI and deep learning (DL) are making brachytherapy treatment workflows more efficient, robust performance on post‐catheter CT images remains challenging, particularly when metal needles and applicators introduce streaking and artifacts near the target.^6^

Most prior AI autosegmentation studies in brachytherapy have concentrated on gynecologic sites and often on MRI, with fewer studies addressing post‐catheter CT in prostate HDR settings. For example, Li et al used a self‐adapting nnU‐Net ensemble in gynecologic HDR brachytherapy to auto‐segment bladder, rectum, and HR‐CTV on CT, reporting strong geometric performance while also noting dosimetric differences.^7^ Xue et al. further explored a 3D Prompt‐nnU‐Net for postoperative endometrial carcinoma, demonstrating good geometric performance across the HR‐CTV and OARs on CT.^8^ Studies that have investigated AI contouring for HDR prostate brachytherapy have primarily used transrectal ultrasound (TRUS) images.^9^, ^10^, ^11^, ^12^, ^13^, ^14^ Krupien et al.^15^ evaluated open‐source DL models for both prostatic and gynecological HDR brachytherapy contouring but only reported results for rectum and bladder, and did not include a dosimetric evaluation. Despite these advances, there remains a need for a CT‐based model capable of contouring the target and all relevant nearby anatomy for HDR prostate brachytherapy.

To address this gap, we developed and validated an autosegmentation model for CT‐based HDR prostate brachytherapy planning using a self‐configuring, open‐source DL framework. Our approach leverages the nnU‐Net architecture, trained from scratch on a curated dataset of clinically approved contours, to provide a robust solution for prostate and OAR segmentation^16^. This work aims to facilitate broader adoption of AI‐driven tools in brachytherapy workflows, improving efficiency, consistency, and treatment quality.

## 2 METHODS

### 2.1 Image acquisition and preprocessing

The images used in this work were collected retrospectively as part of an IRB‐approved study (#2024–0218) from 242 patients who underwent HDR prostate BT in conjunction with EBRT. Inclusion criteria consisted of availability of post‐catheter CT imaging and clinically approved organ segmentations.

Post‐catheter CT images were acquired after Foley catheter and plastic interstitial catheter placement using the NeuroLogica BodyTom 64‐slice mobile CT scanner (NeuroLogica Corp., Danvers, MA). For CT acquisition, brass dummy wires were placed inside each plastic needle for ease of reconstruction and tip identification. CT scans were acquired at 120 kVp and 275 mAs, with a slice thickness of 1.25 mm and in‐plane pixel spacing of 0.7 mm x 0.7 mm. Images were reconstructed using a soft tissue reconstruction kernel and were used to verify needle placement and for CT‐based treatment planning.

Manual segmentations of the prostate, rectum, bladder, and urethra visualized by the Foley catheter without additional margin were retrospectively obtained from clinically approved contours generated by the treating physician for each patient as part of routine clinical practice. Across the full dataset, contours were contributed by two treating physicians. The target volume was defined on a patient‐specific basis and consisted of the prostate without additional margin and optional inclusion of proximal seminal vesicles and/or adjacent periprostatic tissues when clinically indicated. All CT volumes and corresponding segmentations were anonymized and exported in DICOM format and converted to NIfTI for processing.

Preprocessing followed the nnU‐Net 3D full‐resolution pipeline. CT intensities were normalized using CT‐specific z‐score normalization. All images and labels were resampled to a target voxel spacing of 0.7 mm isotropic in‐plane with 1.25 mm slice thickness using spline interpolation for images and nearest‐neighbor interpolation for labels.

### 2.2 Deep learning network architecture

Model training was performed using three nnU‐Net architectures that included a two‐dimensional (2D) U‐Net, a three‐dimensional (3D) full‐resolution configuration, and a 3D U‐Net cascade network that creates a coarse segmentation from a low‐resolution U‐Net followed by refinement using a high‐resolution U‐Net. We chose to utilize the nnU‐Net framework^16^ as it automates and optimizes model parameters by analyzing the input dataset. This innovative approach reduces the time spent on determining these values and designing an architecture best suited for the input data, allowing for faster model development. For the 3D architectures, training was performed on patches of size 192 × 192 × 64 with a batch size of 2. The 2D model was trained on patches of size 192 × 192 with a batch size of 12. Data augmentation included spatial transformations and intensity augmentations as implemented in nnU‐Net.

Each model was optimized using the composite Dice + cross‐entropy loss function, which balances region overlap accuracy with voxel‐wise classification performance. This combination mitigates class imbalance and improves convergence stability for small structures such as the urethra. Optimization was performed using stochastic gradient descent (SGD) with an initial learning rate of 0.01, decayed polynomially over the course of training.

The dataset was randomly split into 206 training (85%) and 36 testing (15%) cases using a random number generator. Training and testing dataset characteristics are summarized in Table 1. Five‐fold cross‐validation was performed on the 206 training cases, where nnU‐Net automatically split the training data into training and validation datasets using an 80:20 split. After each fold was trained, nnU‐Net averages the softmax predictions of the 5 models to create one final “ensemble” model to be used for inference on an independent, unseen set of 36 test cases. Following inference, nnU‐Net performs automated post‐processing to remove holes within the final segmentations. The results presented in this work reflect the outputs generated on the testing dataset using the ensemble method. Training was performed on a NVIDIA RTX 4090 24 GB GPU, using nnU‐Net v2.5.0 and Python v3.10.

**TABLE 1:** Characteristics of training and testing datasets.

	Training	Testing
Number of patients	206	36
Total 2D CT slices	39,001	6,664
Inserted catheters		
Minimum	13	14
Mean	16.8	16.9
Maximum	22	22
Prostate volume (cm^3^)		
Minimum	21.7	22.0
Mean	42.9	42.2
Maximum	95.4	102.9

### 2.3 Reference autosegmentation method

To compare our DL method trained on brachytherapy specific images versus a general‐purpose EBRT technique, we evaluated Contour ProtégéAI (MIM Software Inc., Cleveland, OH) which is a commercially available DL‐based auto segmentation solution integrated within the MIM Maestro platform. Contour ProtégéAI employs a 3D convolutional neural network based on the U‐Net architecture and was trained on a large, multi‐institutional dataset to generate OAR contours.^17^ The system was designed primarily for external beam radiotherapy workflows and does not provide urethra segmentation as part of the “Pelvis CT” auto segmentation site algorithm available at our institution. After the contours were created by Contour ProtégéAI, a post‐processing routine was run to smooth the contours and fill holes. This was accomplished by using the “Smooth” tool, followed by the “Fill Holes…” tool, and a final Smooth″ operation applied to each contour.

### 2.4 Performance evaluation

To assess the quality of the DL‐based segmentations, each of the segmented target and OAR structures were converted into a DICOM RT structure set format. No additional post processing was applied to the model output. Segmentation accuracy was quantitatively evaluated versus the approved reference clinical contours using both geometric and dosimetric metrics.

#### 2.4.1 Geometric evaluation

Segmentation accuracy was assessed for each of the DL methods versus the clinical contours in terms of the dice similarity coefficient (DSC), the 95^th^ percentile Hausdorff distance (HD95%), and the average surface distance (ASD).^18^ The purpose of HD95% and ASD metrics were to quantify the worst case and typical, respectively, boundary discrepancies that are important in brachytherapy plan evaluation. All metrics were computed using the open‐source, PyTorch based MONAI (Medical Open Network for AI) framework to promote reproducibility.^19^ Each metric was computed globally across the entire CT image volume, and also within a restricted volume‐of‐interest (VOI) limited to axial slices containing the clinical planning target volume.^15^ The purpose of the restricted VOI evaluation was to assess the segmentation accuracy in regions most relevant to treatment planning. This is particularly important given that the reference clinical contours used for model training were often incomplete in regions far from the target volume, as these areas were deemed unnecessary for brachytherapy treatment planning and may be omitted to reduce contouring time. Associations between catheter number and DSC were also evaluated using Spearman rank correlation to determine whether segmentation accuracy was impacted by the number of implanted catheters.

#### 2.4.2 Dosimetric evaluation

A dosimetric evaluation was performed to assess the suitability of DL‐based contouring for brachytherapy treatment planning. Each patient included in model training and testing had previously been treated to 15 Gy in a single fraction by interstitial HDR prostate brachytherapy using an Ir‐192 source. The clinically approved dwell positions and dwell times were imported into a previously validated, in‐house TG‐43 based treatment planning system, and dose was re‐computed for each of the three structure sets: nnU‐Net, Contour ProtégéAI, and the reference clinical contours.^20^, ^21^ An isotropic dose grid with 0.5 × 0.5 × 0.5 mm voxels was used for all calculations.

Dose volume histogram (DVH) parameters were computed for OARs, including the minimum dose received by the most irradiated 1 cm^3^ (D1cc) of rectum and bladder, and 0.1 cm^3^ (D0.1cc) of the urethra. Additionally, the percent volume of the prostate PTV receiving 100% of the prescription dose (PTV V100%) was computed. Finally, dosimetric differences were evaluated using clinically relevant parameters prioritized in our HDR prostate brachytherapy workflow, including D1cc, D0.1cc, and PTV V100%, and were computed for each DL segmentation method relative to the reference clinical contours. Difference values close to zero indicate high agreement and support the feasibility of using DL generated contours for brachytherapy treatment planning.

#### 2.4.3 Statistical analysis

The geometric performance metrics for the nnU‐Net and Contour ProtégéAI methods were evaluated for significance versus one another using paired, two‐sided Wilcoxon signed‐rank tests. A p‐value less than 0.05 was considered statistically significant. The urethra was excluded from geometric comparison as it was not contoured by Contour ProtégéAI. Dosimetric endpoints were evaluated for each method relative to the clinical reference contours using paired, two‐sided Wilcoxon signed‐rank tests. To account for multiple comparisons, a Bonferroni‐corrected p‐value of 0.025 was applied to determine statistical significance for the dosimetric analyses.

## 3 RESULTS

### 3.1 Geometric evaluation

**FIGURE 1:**
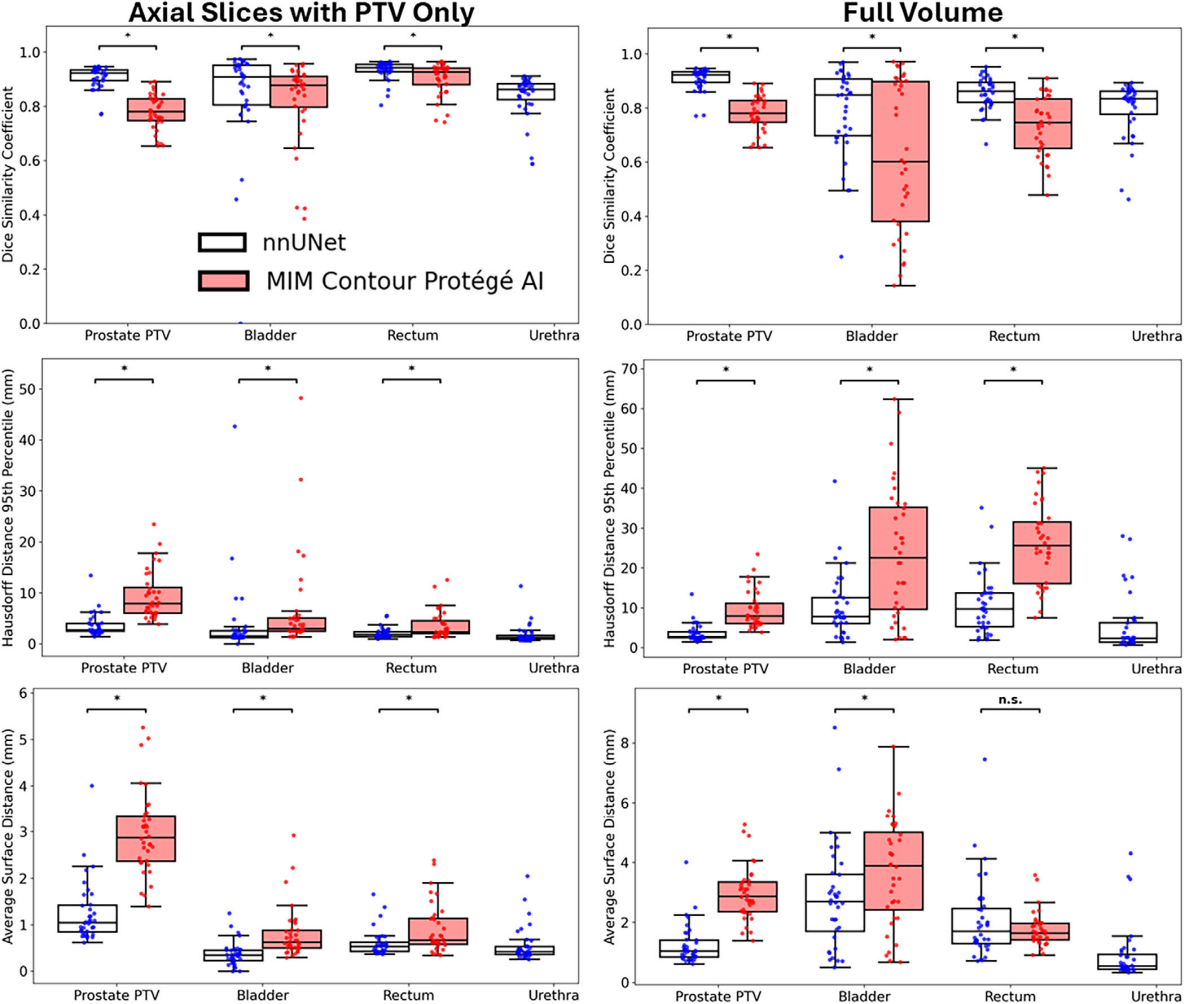
Auto‐contouring performance is presented in terms of DSC (top row), 95^th^ percentile Hausdorff distance (middle row), and ASD (bottom row) for analysis constrained to slices containing the prostate PTV (left column) and for the entire volume (right volume). Significant (*p* < 0.05) differences between the nnU‐Net and ProtégéAI model are marked with an asterisk (*).

Auto‐contouring results are summarized in Figure 1 for the nnU‐Net and ProtégéAI models. A statistically significant improvement in evaluation metrics (DSC, HD95%, ASD) was observed for prostate PTV, bladder, and rectum for the nnU‐Net trained on brachytherapy specific images versus the ProtégéAI model for the analysis limited to axial slices containing prostate PTV. For the full volume analysis, a significant improvement was observed for all metrics and structures except for the rectum ASD for which no significant difference existed between nnU‐Net and ProtégéAI. Table 2 presents the mean +/− standard deviation of the DSC, HD95%, and ASD for the target prostate PTV and OARs. A weak negative correlation was observed between number of catheters and prostate PTV DSC (Spearman *ρ* = −0.35) as shown in Figure S1. No significant (*p* > 0.05) correlations were observed for rectum (*ρ* = −0.17, Figure S2), bladder (*ρ* = −0.17, Figure S3), or urethra (*ρ* = −0.14, Figure S4).

**TABLE 2:** Auto‐contouring results. Prostate PTV, bladder, rectum, and urethra are compared for a network trained on BT planning images (nnU‐Net) and a commercial EBRT method (ProtégéAI) versus clinical segmentations. Results are presented as the mean +/− standard deviation for a VOI containing the prostate PTV and for the full volume.

Structure	Model	DSC	HD95% (mm)	ASD (mm)
Prostate PTV	nnU‐Net	0.91 +/− 0.04	3.62 +/− 2.23	1.25 +/− 0.66
	ProtégéAI	0.78 +/− 0.07	9.52 +/− 4.73	2.97 +/− 0.90
Bladder	nnU‐Net (PTV Extent)	0.85 +/− 0.19	3.73 +/− 7.34	0.37 +/− 0.26
	ProtégéAI (PTV Extent)	0.82 +/− 0.15	6.55 +/− 9.39	0.81 +/− 0.55
	nnU‐Net (Full Volume)	0.79 +/− 0.16	10.19 +/− 8.01	2.83 +/− 1.75
	ProtégéAI (Full Volume)	0.63 +/− 0.28	23.53 +/− 16.47	3.66 +/− 1.75
Rectum	nnU‐Net (PTV Extent)	0.93 +/− 0.04	2.08 +/− 1.03	0.60 +/− 0.29
	ProtégéAI (PTV Extent)	0.90 +/− 0.06	3.61 +/− 2.70	0.90 +/− 0.54
	nnU‐Net (Full Volume)	0.86 +/− 0.06	10.76 +/− 7.45	2.07 +/− 1.33
	ProtégéAI (Full Volume)	0.74 +/− 0.11	26.15 +/− 10.20	1.76 +/− 0.58
Urethra	nnU‐Net (PTV Extent)	0.84 +/− 0.09	1.83 +/− 1.92	0.56 +/− 0.38
	nnU‐Net (Full Volume)	0.80 +/− 0.10	5.53 +/− 7.30	0.92 +/− 0.92
	ProtégéAI	–	–	–

**FIGURE 2:**
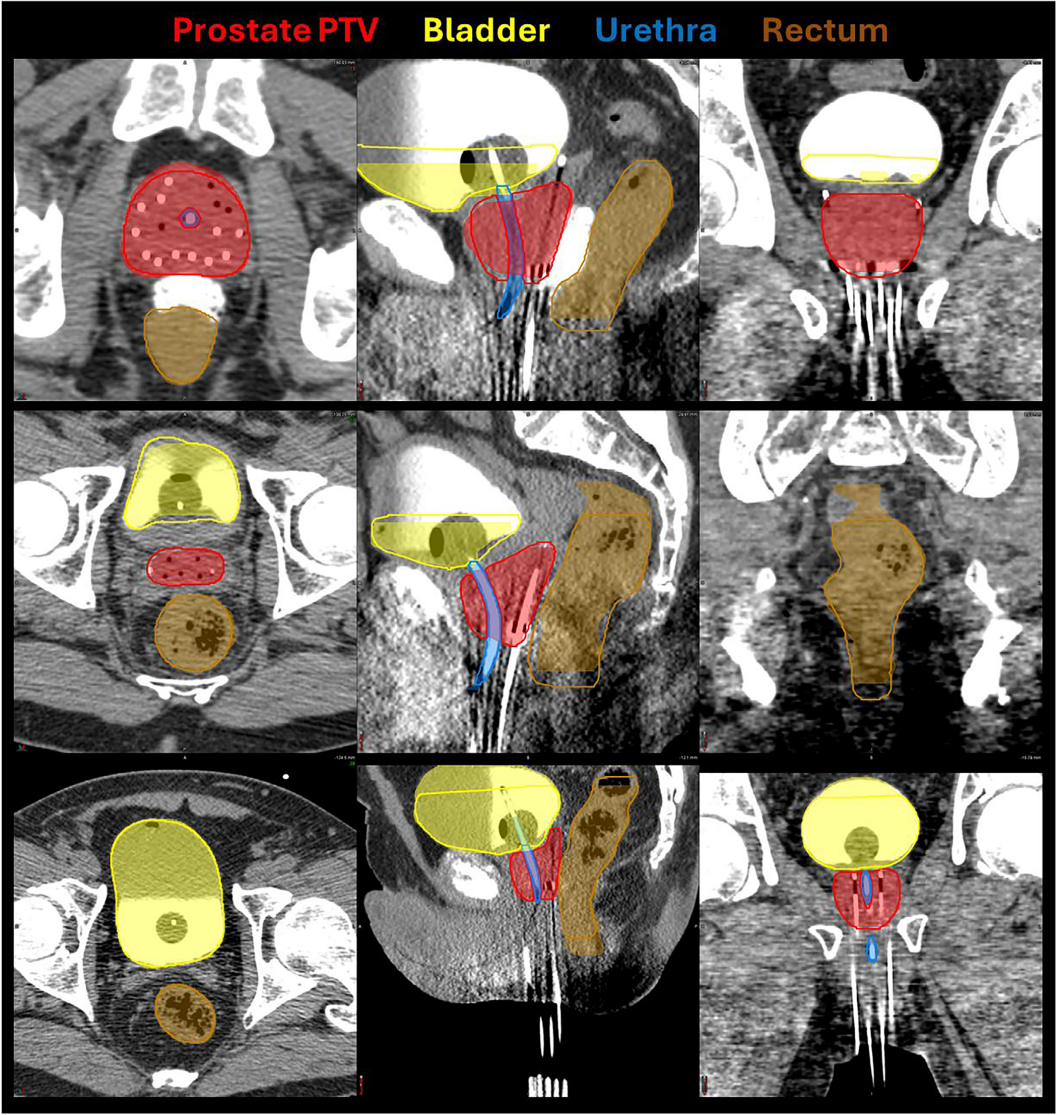
Typical segmentations assessed in terms of the median DSC for prostate (top row), rectum (middle row), and bladder (bottom row) are presented for the DL technique (thick lines, unfilled) versus clinical contours (thin lines, 40% fill).

**FIGURE 3:**
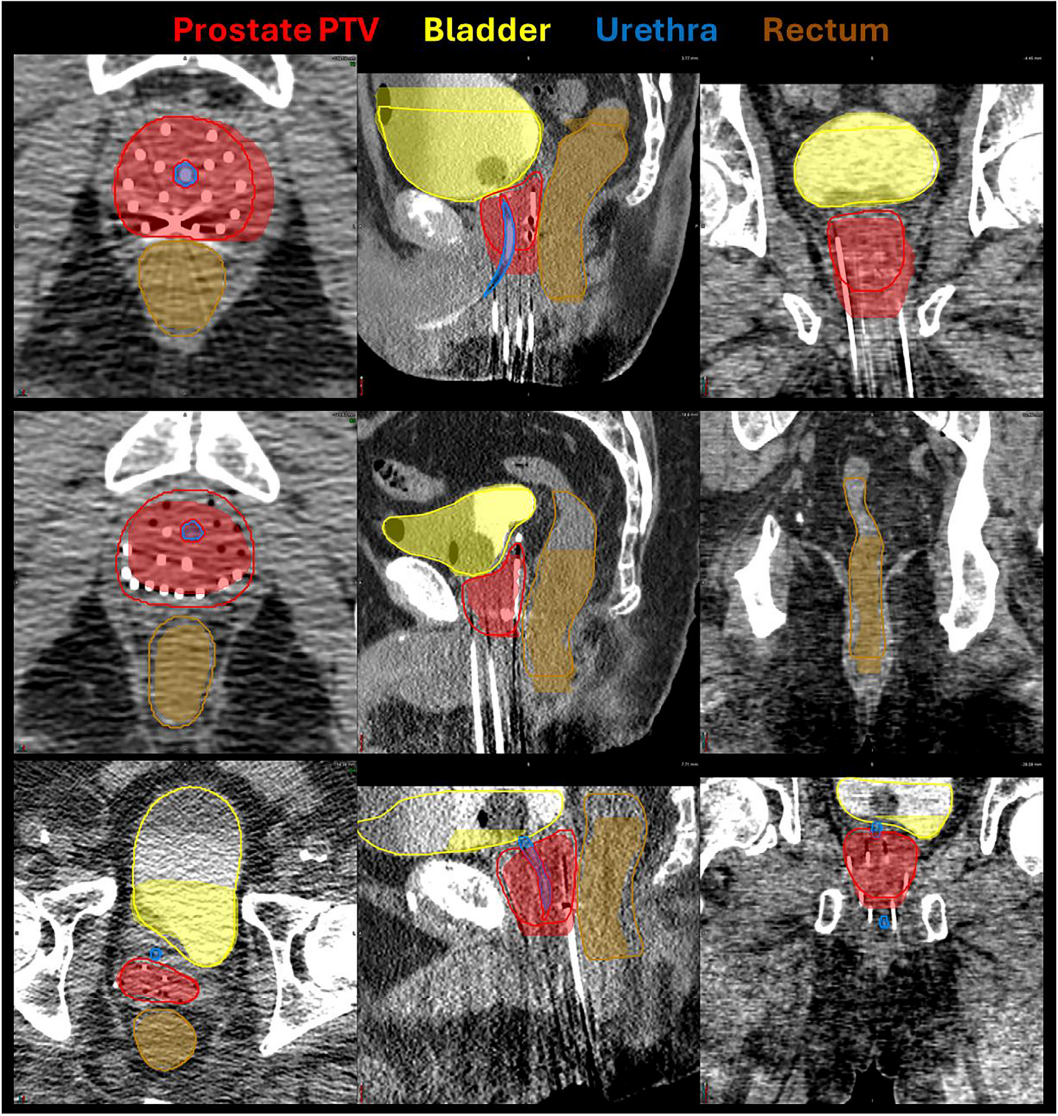
Worst case performance segmentations assessed in terms of the minimum DSC are presented for prostate and rectum (top row) and bladder (bottom row). The DL contours are presented as unfilled with thick lines. The clinical contours are presented with 40% fill.

Example segmentations representative of expected performance are presented in Figure 2 overlaid with the clinical contours. The nnU‐Net segmentations are presented for testing set cases corresponding to the median DSC for the prostate PTV, rectum, and bladder. Worst case performance in terms of the minimum DSC is demonstrated in Figure 3. For the minimum DSC prostate case (Figure 3, top row), the clinical contour extends further laterally from the needles, relative to the DL contour, and median performance contour demonstrated in Figure 2. For the minimum DSC rectum case (Figure 3, middle row), three slices that appear to be rectum within the axial planes containing prostate PTV were not contoured clinically. Similarly, the clinical bladder contour consists of only a few slices for the minimum bladder DSC (Figure 3, bottom row) case. These examples demonstrate how the developed technique could be used either as the primary approach to automate contouring and improve procedure efficiency, or else as a quality assurance tool during the initial plan check.

### 3.2 Dosimetric evaluation

**FIGURE 4:**
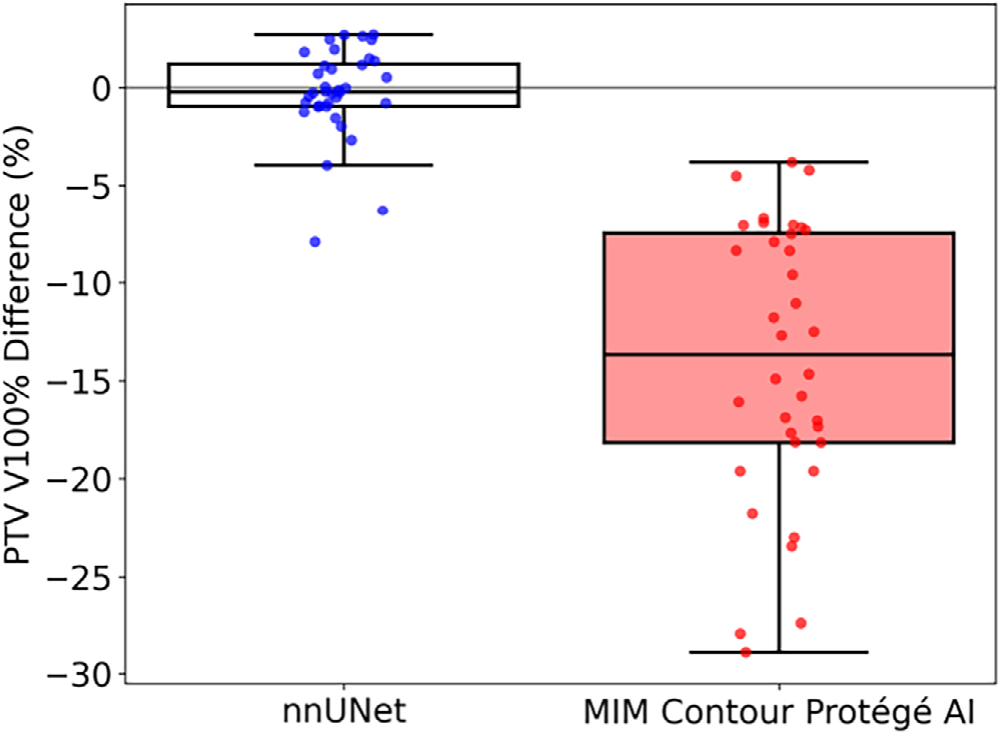
The difference in the percent volume of the prostate PTV receiving 100% of the prescription dose is shown for the nnU‐Net trained on brachytherapy specific planning images and a commercial method used for EBRT male pelvis contouring.

**FIGURE 5:**
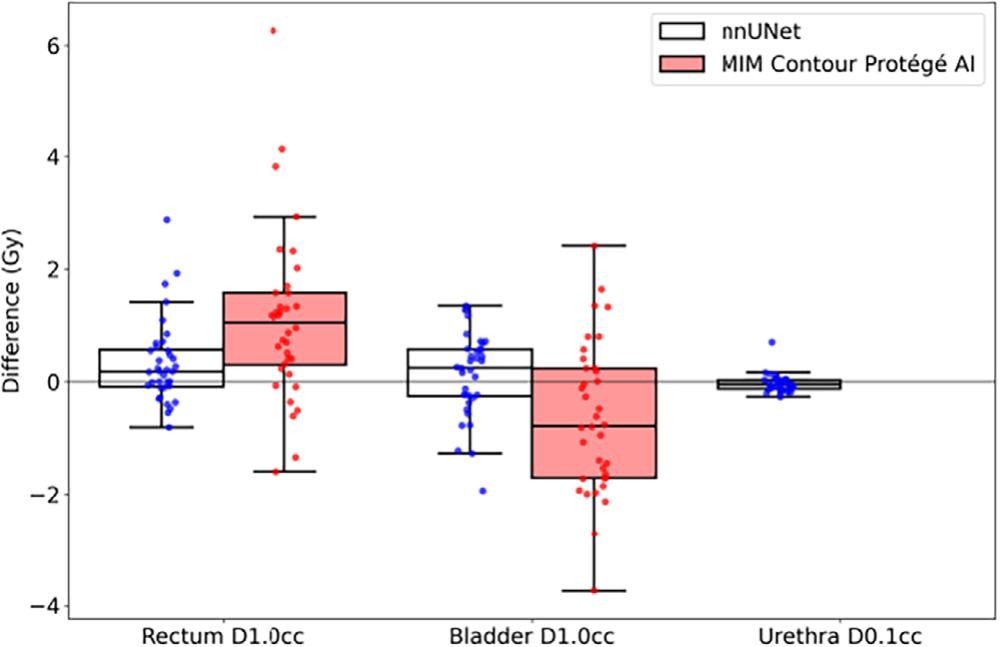
The difference in dose (Gy) between DL and physician contours is shown for brachytherapy relevant dosimetric endpoints for rectum, bladder, and urethra.

To evaluate the suitability of DL contours for brachytherapy treatment planning, the differences in dosimetric endpoints were computed between each of the DL networks (nnU‐Net and ProtégéAI) and the clinical contours. Figure 4 presents the differences in prostate PTV V100%. No statistically significant differences were observed for PTV V100% for the nnU‐Net (*p* = 0.91) versus the clinical contours. ProtégéAI differences were significant (*p *= 2.9E‐11). Figure 5 displays the dose differences for each of the OARs. All differences were significant with the exception of the bladder D1.0cc dose for the nnU‐Net (*p* = 0.187). Table S1 summarizes the dosimetric differences.

## 4 DISCUSSION

Accurate auto‐contouring of target volumes and OARs in brachytherapy has the potential to reduce procedure and anesthesia times and to enable more timely feedback for catheter repositioning or placement of additional catheters to improve treatment plan quality. Automated segmentation may also reduce variability, improve contour consistency, or be used as a quality assurance tool during initial plan review to enhance patient safety. While DL‐based autosegmentation has been widely adopted in EBRT, its application to brachytherapy remains limited. Brachytherapy presents unique contouring challenges due to steep dose gradients and the presence of radiopaque markers within the patient, making contour accuracy particularly critical at organ boundaries adjacent to or abutting the target. Although several prior studies have demonstrated promising DL segmentation performance for gynecological brachytherapy, these approaches are not readily transferable to prostate HDR brachytherapy, which introduces additional complexities including a high number of interstitial catheters, artifact producing catheter markers and gold fiducials, and the presence of a Foley catheter and bladder contrast. The objective of this work was to implement and evaluate a DL‐based auto‐contouring approach specifically for post‐catheter CT imaging in HDR prostate brachytherapy.

To the best of our knowledge, this work presents the first DL‐based segmentation method to automatically contour the prostate PTV and urethra via Foley catheter on post‐catheter CT images for HDR prostate brachytherapy. In addition, we report the first evaluation of a DL model trained exclusively on prostate brachytherapy CT images to segment the prostate PTV, urethra, bladder, and rectum. To date, only one prior study has evaluated DL contouring for prostate brachytherapy; however, that model was trained using a combined cohort of prostate and gynecological brachytherapy patients and was limited to segmentation of the bladder and rectum, without inclusion of the prostate target or urethra. ^15^ Importantly, the present study also provides the first evaluation of differences in clinically relevant dosimetric endpoints when HDR prostate brachytherapy planning is performed using DL contours compared with clinically approved contours. An additional contribution of this work is the use of a fully 3D nnU‐Net architecture for prostate brachytherapy segmentation, whereas the study by Krupien et al.^15^ was restricted to a 2D implementation due to GPU constraints. Finally, our DL model was benchmarked against a commercially available DL segmentation algorithm developed for external beam radiotherapy of the male pelvis to assess whether models designed for EBRT can achieve acceptable performance in the brachytherapy setting. However, differences in the training data and intended clinical application should be considered when interpreting these results, as the commercial model was not trained on post‐catheter CT images and likely did not include brachytherapy specific artifacts or the same target and organ contouring extents.

Our DL nnU‐Net provided excellent target segmentation accuracy, with a mean DSC of 0.91 +/− 0.04, compared with 0.78 +/− 0.07 for ProtégéAI (Table 2). Similarly, the mean ASD was 1.25 +/− 0.66 mm for nnU‐Net versus 2.97 +/− 0.90 mm for ProtégéAI. Despite the excellent DSC, a limitation of our method is that target definitions included the prostate ± seminal vesicles or periprostatic tissues. This variability in target definition may have contributed to differences in contouring performance across patients. The nnU‐Net outperformed the EBRT focused ProtégéAI model across all evaluation metrics except for the rectum ASD evaluated over the full image volume, where ProtégéAI achieved a mean ASD of 1.76 +/− 0.58 mm versus 2.07 +/− 1.33 mm for nnU‐Net. However, when restricting the rectum analysis to the VOI most relevant to brachytherapy, that is, axial image slices containing prostate PTV, the nnU‐Net outperformed ProtégéAI with a mean DSC of 0.93 +/− 0.04 versus 0.90 +/− 0.06 and ASD equal to 0.60 +/− 0.29 mm versus 0.90 +/− 0.54 mm. An additional advantage of the nnU‐Net approach is its ability to segment the urethra, with a mean DSC of 0.84 +/− 0.09 and ASD equal to 0.56 +/− 0.38 mm, whereas ProtégéAI does not provide urethra segmentation. Our results indicate that significant (*p* < 0.05) improvements in performance can be achieved by training a DL network on post‐catheter brachytherapy specific images. While EBRT‐focused DL architectures may yield acceptable performance for certain OARs such as the rectum, our findings demonstrate that improved and more consistent performance is obtained when networks are trained on brachytherapy specific data and contouring workflows, including post‐catheter implant CT images and clinically relevant regions of interest such as OAR and target interfaces. For all organs and evaluation metrics, segmentation performance was reduced when assessed over the full image volume compared with VOI restricted evaluation. This behavior is expected, as the clinically approved contours used for training and evaluation (Figure 2 and Figure 3) are often incomplete at clinically irrelevant distances far from the prostate PTV for the bladder and rectum, highlighting both a unique challenge and important context to consider for auto‐contouring in brachytherapy applications.

As previously noted, this work describes the first DL method to segment all clinically relevant targets and OARs in CT‐based HDR prostate brachytherapy. Consequently, there is limited published data to directly compare our results against. The most closely related study is that by Krupien et al.^15^, which reported median DSC values of 0.96 for the bladder (compared with 0.91 in the present work) and 0.88 for rectum (compared with 0.94 in the present work) when analysis was restricted to axial slices containing the CTV, using a large mixed dataset of prostate and gynecological HDR brachytherapy patients.

While geometric evaluation is an important consideration, dosimetric differences are perhaps more relevant in the brachytherapy setting due to the potential for incomplete organ contouring. As shown in Figure 4, our method resulted in only minor dosimetric differences in target coverage, as assessed by PTV V100%. The mean difference in PTV V100% was −0.25 +/− 2.30% for nnU‐Net versus −13.98 +/− 7.04% for ProtégéAI. The maximum difference observed for our method was −7.9%. The difference in bladder and rectum D1.0cc was 0.13 +/− 0.75 Gy and 0.34 +/− 0.74 Gy, respectively, for nnU‐Net, versus −0.65 +/− 1.33 Gy and 1.11 +/− 1.51 Gy for ProtégéAI. The difference in D0.1cc for the urethra was −0.03 +/− 0.16 Gy for the nnU‐Net. The observed mean difference in PTV V100% of −0.25% is likely clinically negligible, suggesting that nnU‐Net derived prostate PTV contours could be used following physician review without modification for the majority of patients to improve procedural efficiency. Similarly, mean dosimetric differences for all nnU‐Net OAR segmentations were small (< 0.4 Gy), indicating accuracy suitable for treatment planning, following physician contour review. Several outliers were observed in both the geometric segmentation metrics (Figure 1) and dosimetric analyses (Figures 4 and 5). Visual review indicated that these cases were primarily attributable to challenging imaging conditions and localized contouring disagreements rather than gross segmentation failures. Examples of the worst‐case performance for each contour can be seen in Figure 3. For the prostate PTV, the nnU‐Net generated contours extended further superiorly than the clinical contours. Visual inspection of these cases suggested that the model may have incorporated information from the needle placement geometry to define the extent and boundary of the prostate PTV volume. Overall, this resulted in larger target volumes and thus lower calculated V100% values as shown in Figures S5 and S6. Rectum D1.0cc outliers occurred when the nnU‐Net model delineated the anterior rectal wall closer to the PTV than the clinical contour (Figures S7, S8, S9), causing increased sensitivity in this high dose gradient region. This may be due to the limited soft tissue contrast between the prostate and rectal wall. Image fusion including an MRI was used for the clinical/reference contours which helps define prostate PTV borders. Future work may consider including pre‐procedure MRI, PET/CT, and clinical notes via a LLM (e.g. Vision Mamba^22^) to improve performance. The bladder outlier (Figure S10) appears to have occurred due to poor centering of the CT image, resulting in part of the bladder to be cropped out of the image. Additionally, 1–2 catheters appear to be inserted into or near the bladder wall causing the model to carve this portion out of the bladder contour. Lastly, the urethra outlier (Figure S11) is due to poor Foley catheter visualization on the CT image due to low or absent contrast within the Foley catheter during image acquisition. Future prospective studies are warranted to evaluate the clinical utility of DL based autosegmentation for HDR prostate brachytherapy and to evaluate the time and degree of modifications that may be required for routine clinical deployment.

While the presented results are promising for clinical use, several limitations should be addressed prior to deployment. First, this study was conducted using data from a single institution and a single mobile CT scanner, and inter‐observer variability was not explicitly quantified. This limits the generalizability of our results and the robustness of the final model. Future work is warranted to evaluate model performance across additional institutions, CT scanners, and imaging protocols. Second, our evaluation was limited to a single DL architecture, nnU‐Net, which was selected due to its strong documented performance in radiotherapy applications. ^7^, ^8^, ^15^, ^23^, ^24^ An important area for future investigation will be the evaluation of geometrically focused training strategies to further improve segmentation performance for brachytherapy‐specific use cases.^25^ Third, as previously discussed, the clinically approved bladder and rectum contours used for model training often truncated the organs in the superior or inferior directions at regions distant from the prostate PTV that are not relevant to brachytherapy treatment planning. Notably, the DL model adapted to this characteristic of the training data and produced high quality segmentations in the most clinically relevant regions, while frequently omitting the most superior portions of the bladder or rectum. Nevertheless, future studies could explore retraining the model using a smaller, curated dataset in which full organ volumes are consistently contoured, potentially trading dataset size for annotation consistency. Although contouring times were not prospectively collected in this study, inference and post‐processing using the proposed DL framework were typically completed in less than 30 seconds per case. In contrast, manual contouring requires substantially longer durations. Future prospective studies should formally quantify workflow efficiency and clinician editing time across automated and manual contouring approaches. Finally, hydrogel spacers to separate the prostate and rectum are more commonly being used for prostate patients but were not considered in this work. Future work could consider extending a deep unsupervised clustering technique to improve performance for patients with hydrogel spacer.^26^

## 5 CONCLUSION

We implemented and evaluated the first DL auto‐contouring method for CT‐based HDR prostate brachytherapy. The model successfully contoured the prostate PTV, bladder, rectum, and urethra with high accuracy. Dosimetric differences in target coverage were not statistically significant which supports clinical use. DL based auto‐contouring may improve contouring consistency, procedural efficiency, and reduce anesthesia time in HDR prostate brachytherapy workflows. While DL models developed for EBRT demonstrated utility for segmenting some OARs, our results show that improved accuracy is achieved using a brachytherapy‐specific DL model.

## AUTHOR CONTRIBUTIONS

All authors made substantial contributions to this work and approved the final manuscript.

## CONFLICT OF INTEREST STATEMENT

Dr. Slagowski acknowledges this project was supported in part by the Specialized Program of Research Excellence (SPORE) program, through the National Cancer Institute (NCI), grant P50CA269011. The content is solely the responsibility of the authors and does not necessarily represent the official views of the National Institutes of Health. Additional support for this work was provided by a Badger Challenge grant from the University of Wisconsin‐Madison. The other authors declare no conflicts of interest relevant to this work.

## Supporting information




Supporting Information
