# Supplementary material for: Deep learning auto‐contouring of target and organs‐at‐risk on post‐catheter implant CT images for prostate HDR brachytherapy
Source: J Appl Clin Med Phys. 2026 Aug 18;27(9):e70748. doi: 10.1002/acm2.70748 (PMC13486957; doi:10.1002/acm2.70748)
Supplement: Supplementary file 1 [file ACM2-27-e70748-s001.pdf]

Supplementary Information

Table S1: Dosimetric differences for nnU-Net and ProtégéAI versus clinical contours.

Structure	Model	ΔV100% (%)	ΔD1.0cc (Gy)	ΔD0.1cc (Gy)
Prostate PTV	nnU-Net	-0.25 +/- 2.30	--	--
	ProtégéAI	-13.98 +/- 7.04	--	--
Bladder	nnU-Net	--	0.13 +/- 0.75	--
	ProtégéAI	--	-0.65 +/- 1.33	--
Rectum	nnU-Net	--	0.34 +/- 0.74	--
	ProtégéAI	--	1.11 +/- 1.51	--
Urethra	nnU-Net	--	--	-0.03 +/- 0.16

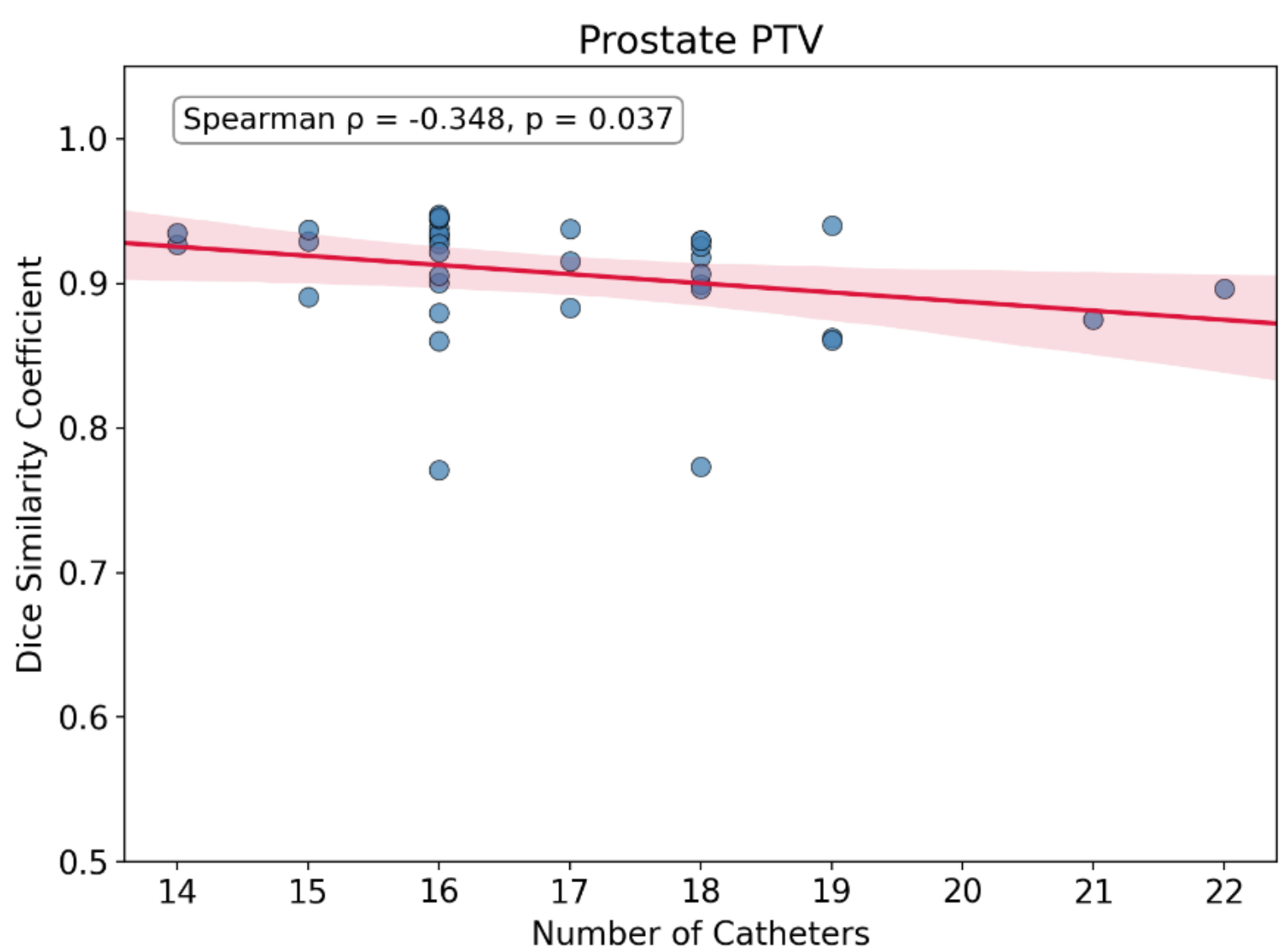

Figure S1: Dice similarity coefficient (full volume analysis) is plotted versus number of catheters for prostate PTV for each individual test case. Statistical significance was evaluated using Spearman rank correlation. The shaded region represents a 95% confidence interval around a linear fit.

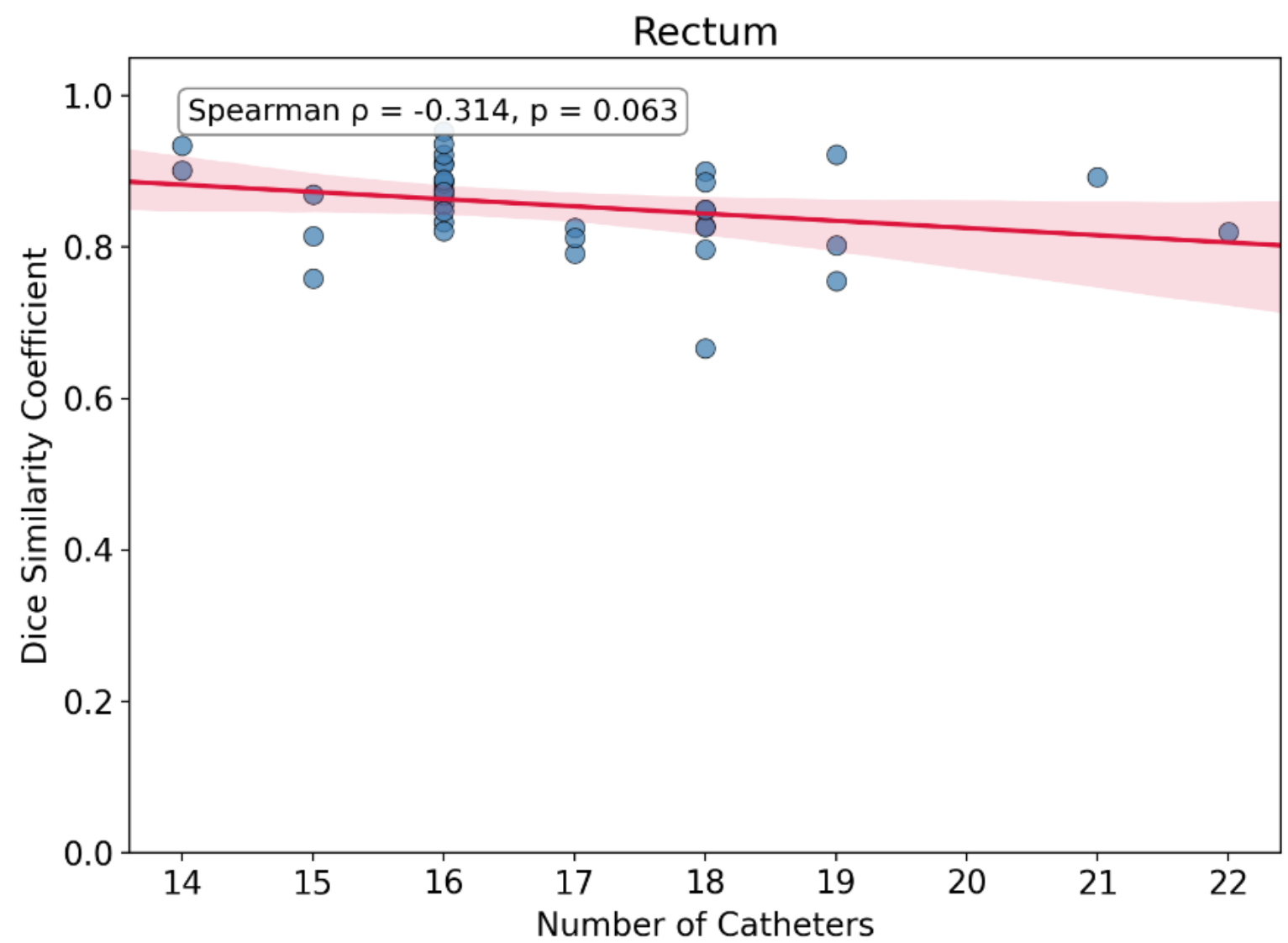

Figure S2: Dice similarity coefficient (full volume analysis) is plotted versus number of catheters for rectum for each individual test case. Statistical significance was evaluated using Spearman rank correlation. The shaded region represents a 95% confidence interval around a linear fit.

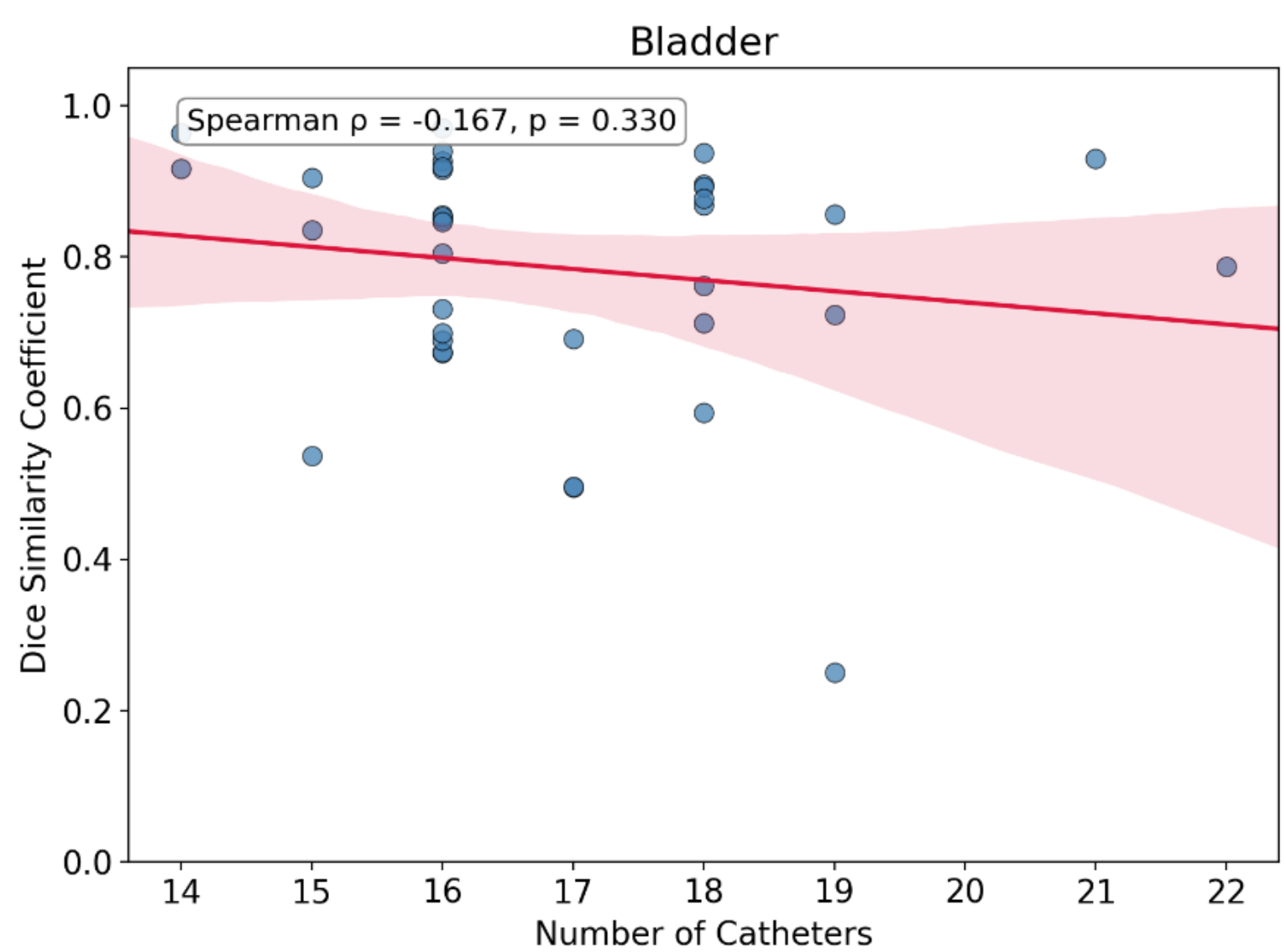

Figure S3: Dice similarity coefficient (full volume analysis) is plotted versus number of catheters for bladder for each individual test case. Statistical significance was evaluated using Spearman rank correlation. The shaded region represents a 95% confidence interval around a linear fit.

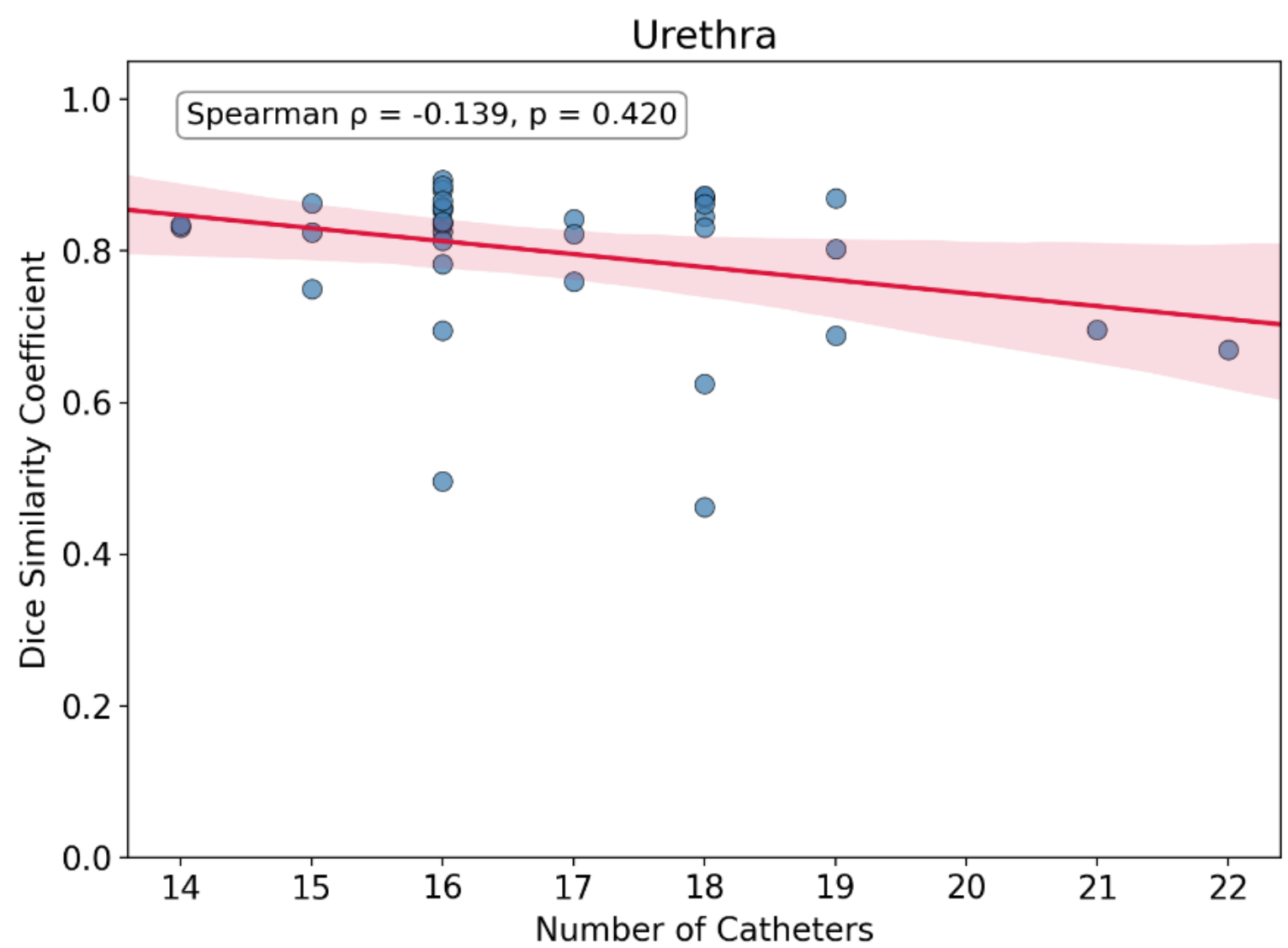

Figure S4: Dice similarity coefficient (full volume analysis) is plotted versus number of catheters for urethra for each individual test case. Statistical significance was evaluated using Spearman rank correlation. The shaded region represents a 95% confidence interval around a linear fit.

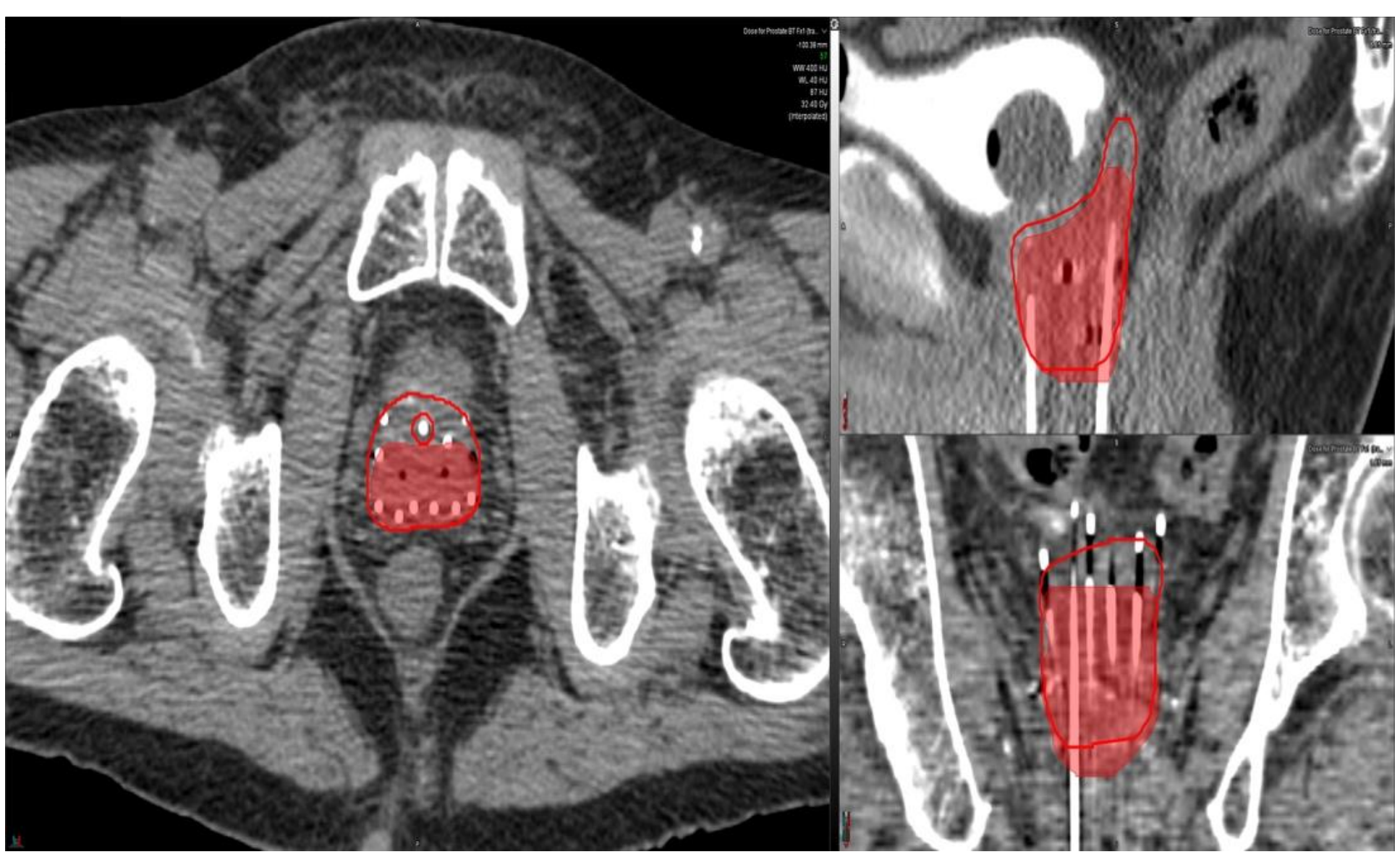

Figure S5: Example of testing subject outlier for Prostate PTV contour comparing deep learning technique (thick lines, unfilled) versus clinical contour (thin lines, 40% fill). Dosimetric difference (V100%) was 7.93% versus clinical contour.

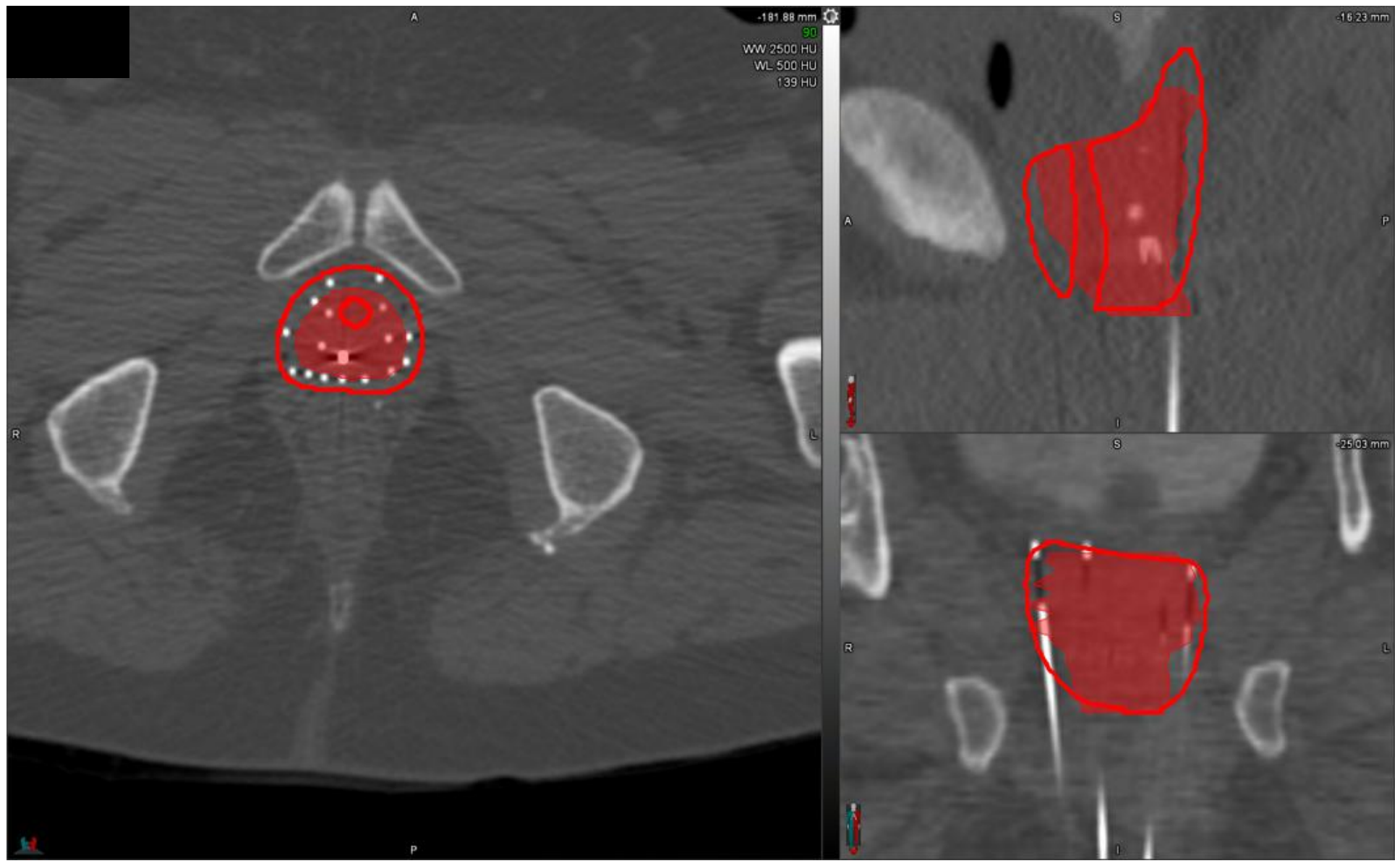

Figure S6: Example of testing subject outlier for Prostate PTV contour comparing deep learning technique (thick lines, unfilled) versus clinical contour (thin lines, 40% fill). Dosimetric difference (V100%) was -6.29% versus clinical contour.

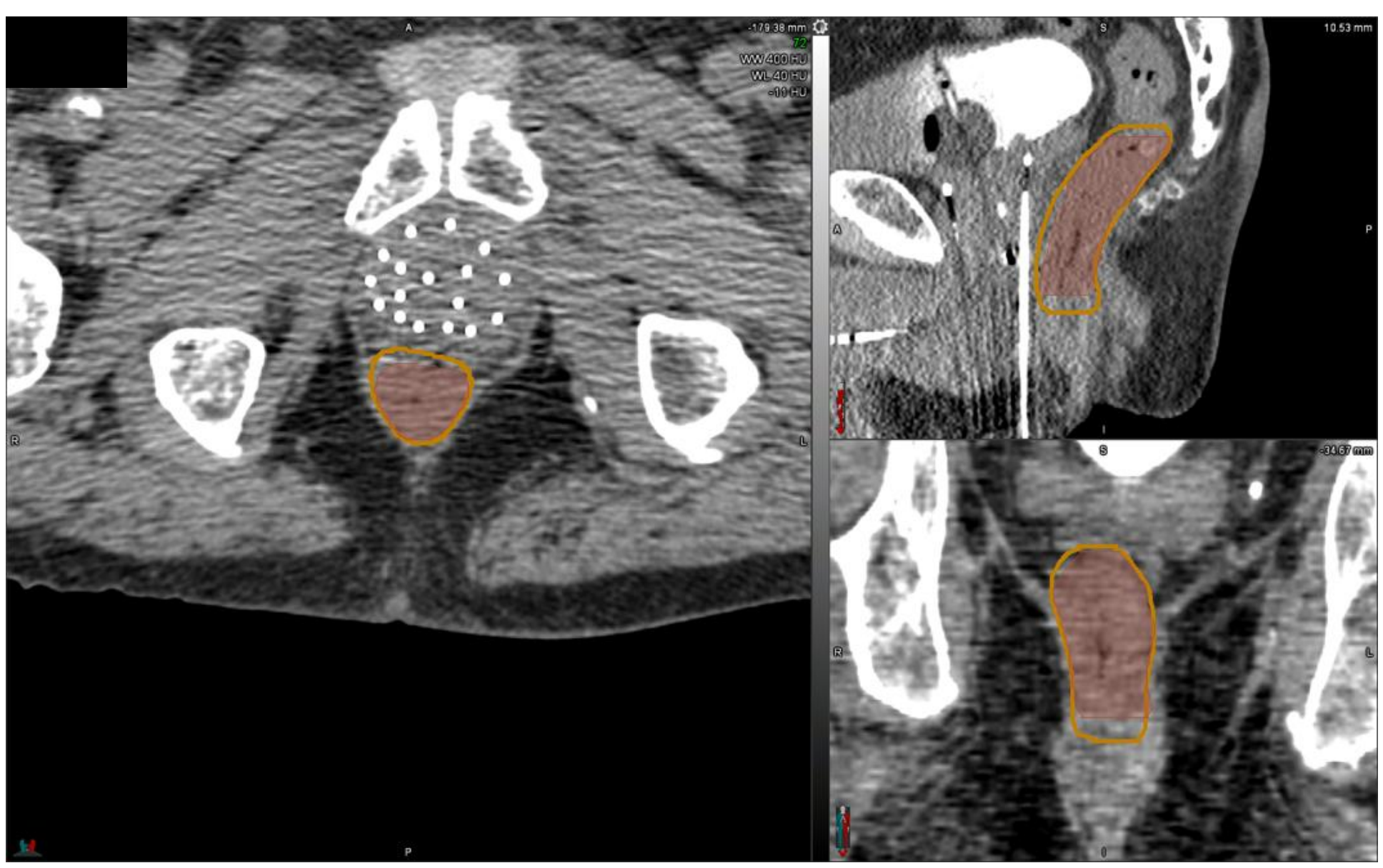

Figure S7: Example of testing subject outlier for rectum contour comparing deep learning technique (thick lines, unfilled) versus clinical contour (thin lines, 40% fill). Dosimetric difference (D1.0cc) was +1.73 Gy versus clinical contour.

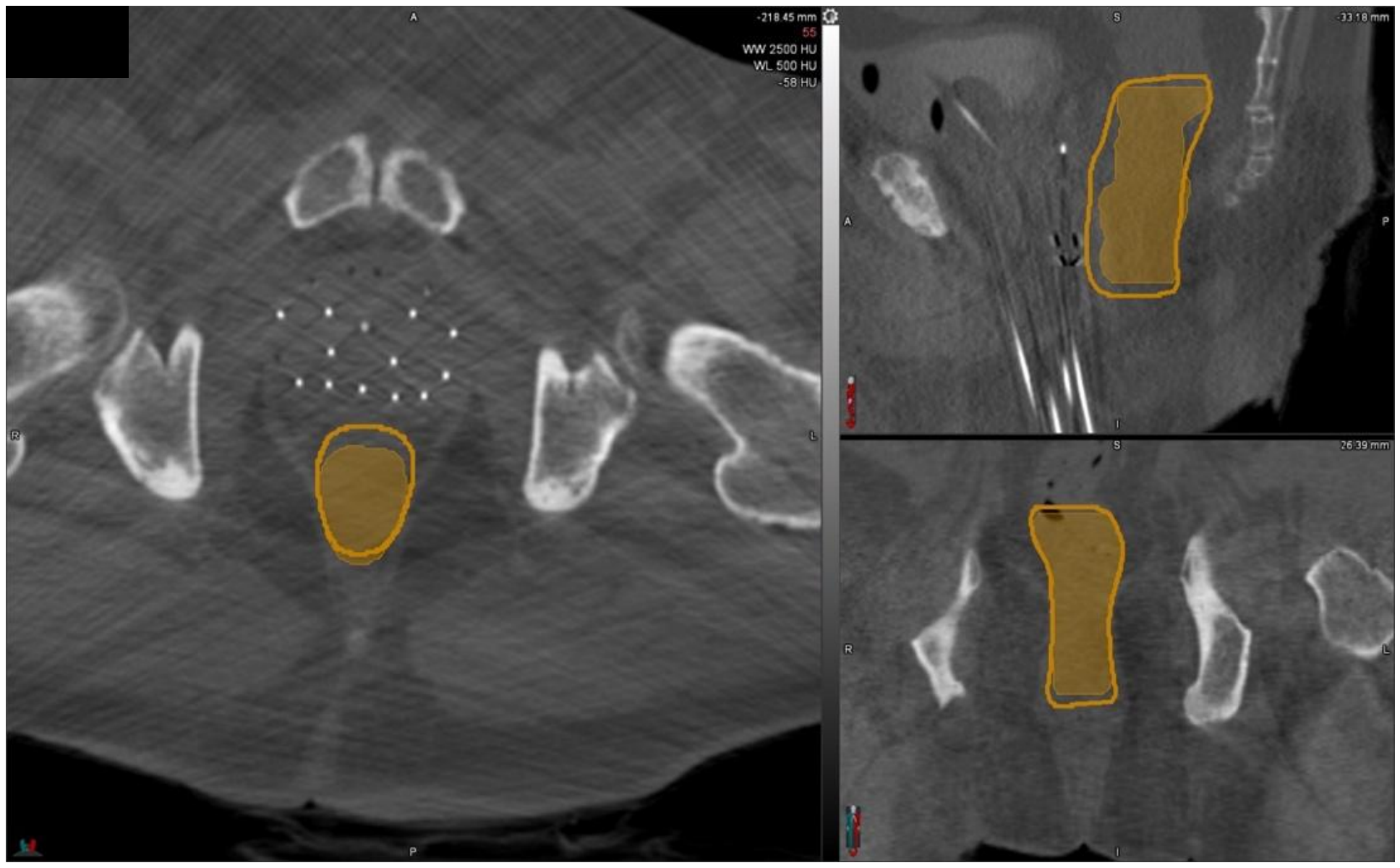

Figure S8: Example of testing subject outlier for rectum contour comparing deep learning technique (thick lines, unfilled) versus clinical contour (thin lines, 40% fill). Dosimetric difference (D1.0cc) was +2.87 Gy versus clinical contour.

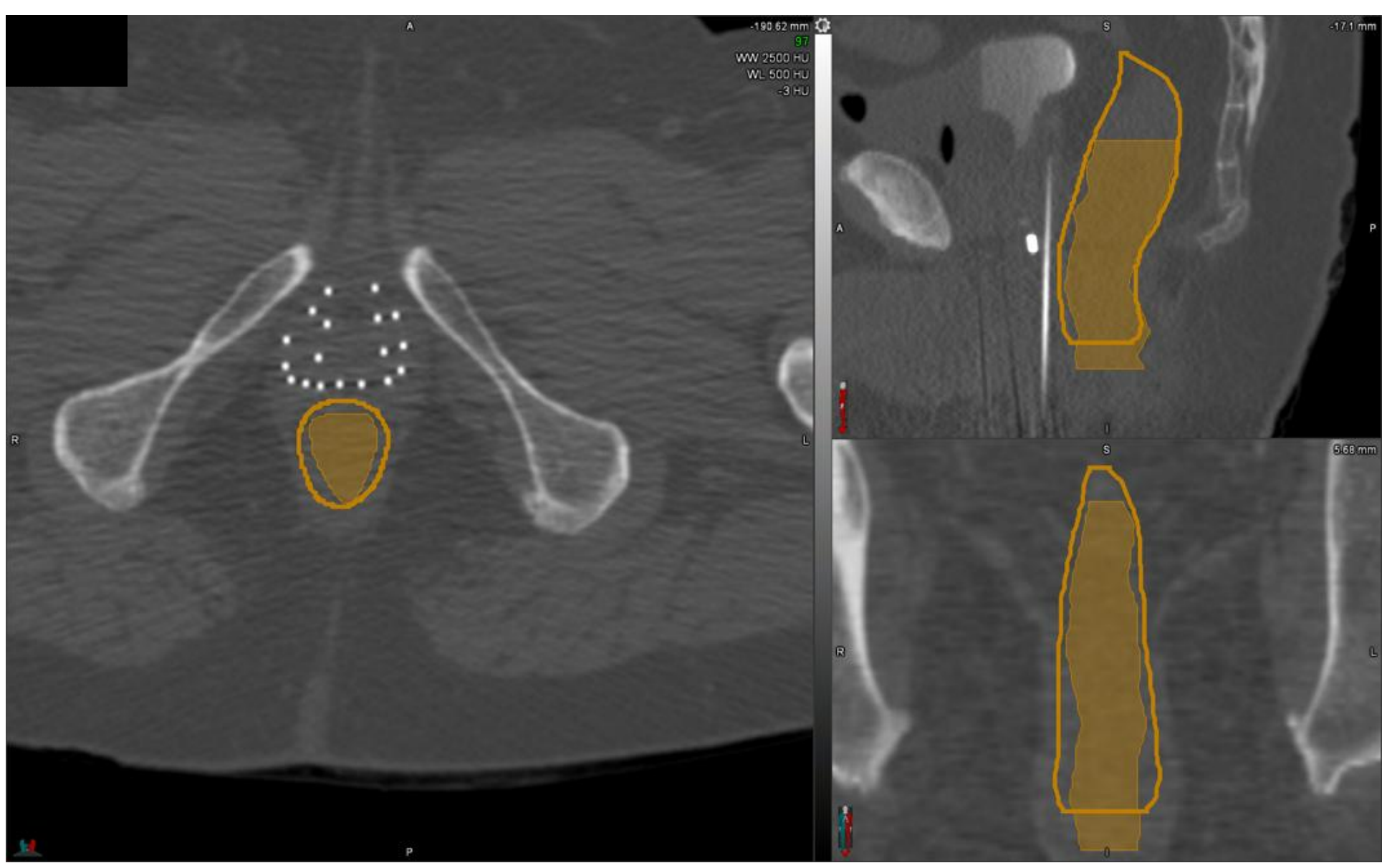

Figure S9: Example of testing subject outlier for rectum contour comparing deep learning technique (thick lines, unfilled) versus clinical contour (thin lines, 40% fill). Dosimetric difference (D1.0cc) was +1.92 Gy versus clinical contour.

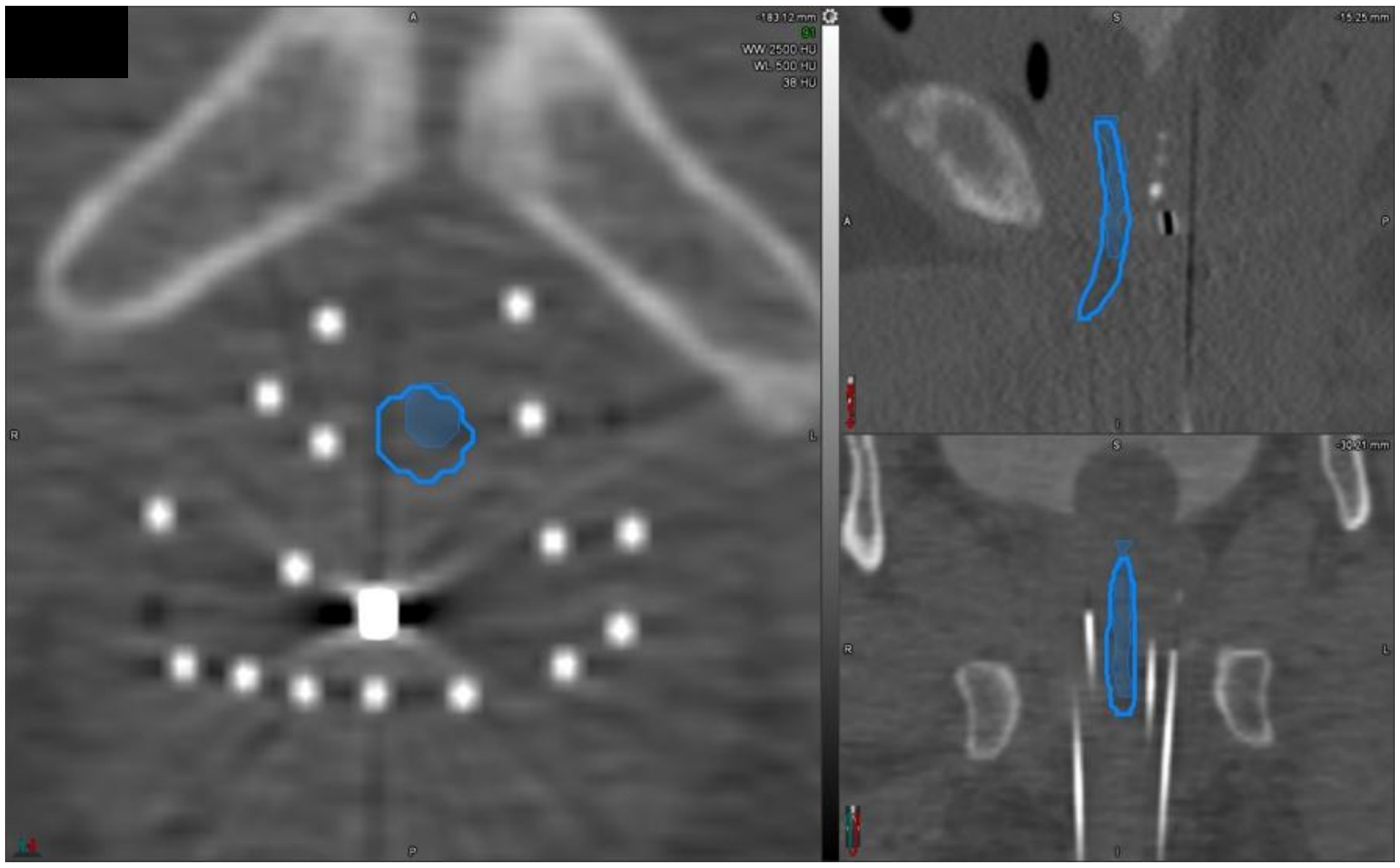

Figure S10: Example of testing subject outlier for urethra contour comparing deep learning technique (thick lines, unfilled) versus clinical contour (thin lines, 40% fill). Dosimetric difference (D0.1cc) was +0.70 Gy versus clinical contour.

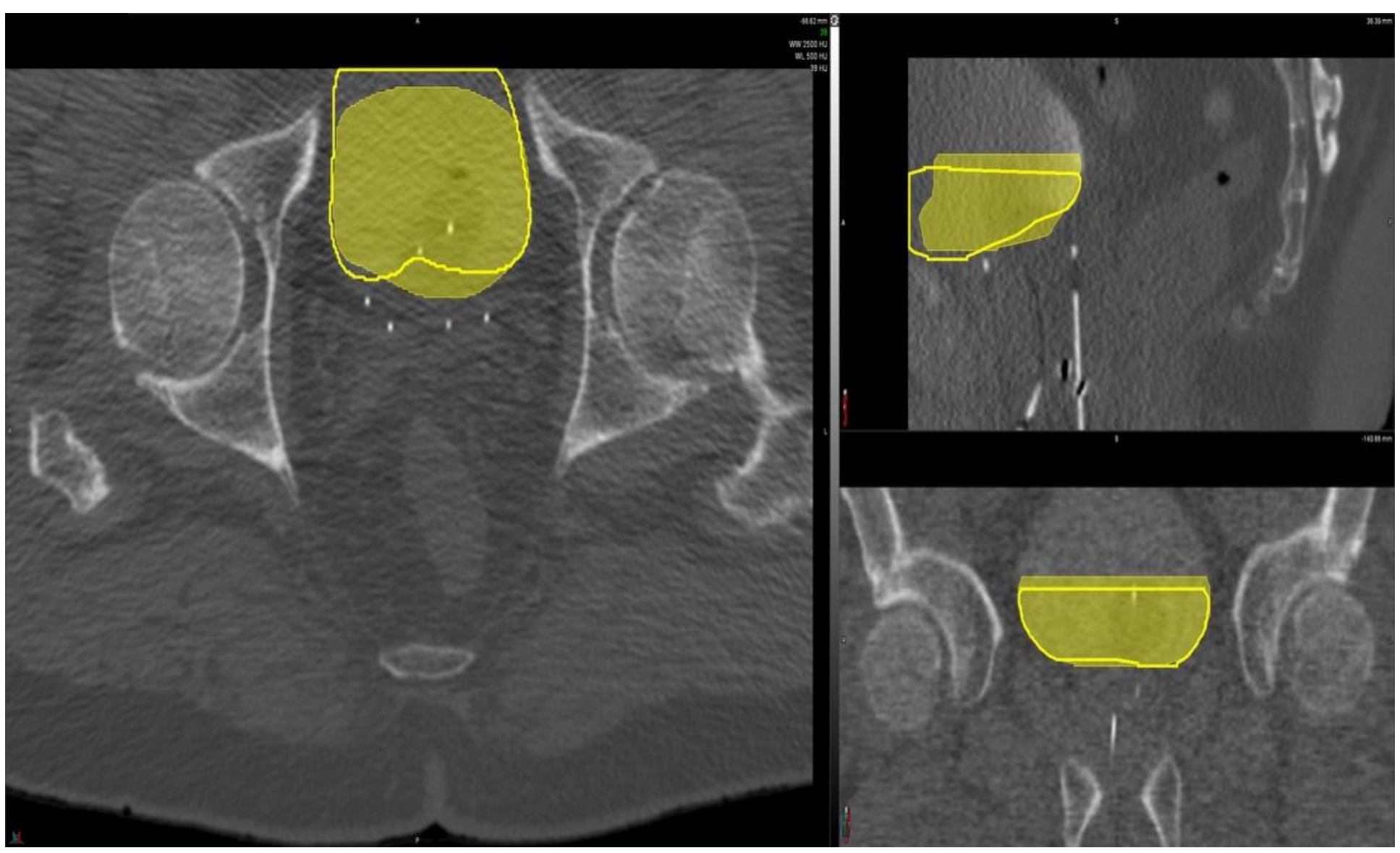

Figure S11: Example of testing subject outlier for bladder contour comparing deep learning technique (thick lines, unfilled) versus clinical contour (thin lines, 40% fill). Dosimetric difference (D1.0cc) was -1.93 Gy versus clinical contour.
